# Designed Ankyrin Repeat Protein (DARPin) to target chimeric antigen receptor (CAR)-redirected T cells towards CD4^+^ T cells to reduce the latent HIV^+^ cell reservoir

**DOI:** 10.1007/s00430-020-00692-0

**Published:** 2020-09-12

**Authors:** Lea Patasic, Janna Seifried, Valerie Bezler, Marcell Kaljanac, Irene C. Schneider, Heike Schmitz, Christiane Tondera, Jessica Hartmann, Andreas Hombach, Christian J. Buchholz, Hinrich Abken, Renate König, Klaus Cichutek

**Affiliations:** 1 Host-Pathogen Interactions, Paul-Ehrlich-Institut, Langen, Germany; 2 Molecular Biotechnology and Gene Therapy, Paul-Ehrlich-Institut, Langen, Germany; 3 Center for Molecular Medicine Cologne, University of Cologne, and Department I of Internal Medicine, University Hospital Cologne, Cologne, Germany; 4 German Center for Infection Research (DZIF), Langen, Germany; 5 Regensburg Center for Interventional Immunology (RCI), Department of Genetic Immunotherapy, University Hospital Regensburg, Regensburg, Germany; 6 Immunity and Pathogenesis Program, Sanford Burnham Prebys Medical Discovery Institute, La Jolla, CA USA; 7 Present Address: Department for Infectious Disease Epidemiology, Robert Koch-Institute, Berlin, Germany

**Keywords:** DARPin, CAR T cells, HIV reservoir, T cell therapy

## Abstract

Chimeric Antigen Receptor (CAR)-redirected T cells show great efficacy in the patient-specific therapy of hematologic malignancies. Here, we demonstrate that a DARPin with specificity for CD4 specifically redirects and triggers the activation of CAR engineered T cells resulting in the depletion of CD4^+^ target cells aiming for elimination of the human immunodeficiency virus (HIV) reservoir.

**Electronic supplementary material:**

The online version of this article (10.1007/s00430-020-00692-0) contains supplementary material, which is available to authorized users.

## Introduction

Engineered T cell therapy utilizing chimeric antigen receptors (CARs) is one of the most promising technologies for the treatment of hematologic malignancies [1, 2]. CARs recognize antigen by binding to cell surface proteins independent of MHC (major histocompatibility complex) presentation and with high specificity, avoiding T cell escape mechanisms based on HLA (human leukocyte antigen) loss utilized by many viruses and tumors. Patient’s T cells are isolated and genetically armed ex vivo with CARs encompassing a binding domain for surface antigens expressed on the targeted cells. Upon antigen binding, an immunological synapse is formed resulting in CAR-mediated recruitment of downstream kinases and finally the release of cytotoxic granules containing perforin and granzyme B, leading to the elimination of the targeted cells. The first clinical trials for CAR T cells were performed with the objective of targeting HIV-infected T cells [3], using a CD4 molecule fused to the Fc receptor γ chain (FcRγ) to redirect T cells to viral gp120 on the cell surface of HIV-infected cells. These first-generation CARs, however, failed to reduce the viral burden in patients for prolonged periods, probably in large part due to a lack of a co-stimulation to sustain CAR T cell persistence. Since then, second-, third- and fourth-generation CARs employ additional intracellular costimulatory domains, rendering them more persistent, less susceptible to exhaustion and equipped with functional capacities [4]. However, using HIV gp120 as a target, CAR T cells will not eradicate the virus completely, due to the presence of latent provirus in cellular reservoirs that persist even under successful anti-gp120 CAR T cell therapy since they do not express viral antigens, making their identification challenging [5]. CD4 serves as cell entry receptor for HIV and is expressed by infected cells during active as well as latent state [5, 6]. Specific targeting of CD4 as a cellular target rather than viral proteins may, therefore, enable depletion of the latent viral reservoir. Most frequently used CAR-binding domains consist of single-chain fragments of variable regions antibodies (scFvs) [2, 7]. As an alternative to antibody-based binding domains, Designed Ankyrin Repeat Proteins (DARPins) also cover a broad spectrum of specificities towards potential target antigens. DARPins are derived from ankyrin repeat proteins which are among the most common structural binding motifs in nature and can be selected to bind a given target protein with high affinity and specificity, to antigens that are expressed even at very low levels on the cell surface [8]. DARPins are characterized by high thermodynamic stability [9], consist of one polypeptide chain that forms a functional binding domain. While the low biophysical stability of scFvs could potentially lead to poor surface expression of the CAR, DARPins with higher stability might be advantageous in that respect. Moreover, in contrast to DARPins, scFvs tend to self-aggregate [10] which can lead to spontaneous CAR clustering and T cell activation in the absence of target cells, providing a so-called increased “tonus”. Here, we asked whether the CD4-specific DARPin_57.2, which has previously been shown to inhibit binding of HIV to its cell entry receptor CD4 [11], can be used likewise as an anti-CD4 scFv to redirect CAR T cells towards the CD4^+^ T cell compartment as a proof of concept for eliminating HIV-infected cells, including those harboring the latently infected cell reservoir. We demonstrate efficient and specific depletion of autologous and heterologous CD4^+^ T cells, including latently HIV-infected J-Lat cells, using DARPin-based CAR T cells in vitro paving the way for controlling the virus reservoir in the long-term.

## Materials and methods

### Ethics statement

Buffy-coats obtained from anonymous blood donors were purchased from the “German Red Cross Blood Donor Service Baden-Württemberg Hessen” and “Transfusionsmedizin Universitätsklinik Regensburg”. Due to the use of anonymous samples, ethical approval was not needed according to German guidelines. The research was performed according to the principles expressed in the “Declaration of Helsinki”.

### Cloning

Moloney murine leukemia virus (MoMLV) expression plasmid has been described previously [12]. Restriction sites for single cutting enzymes were inserted by PCR and the amplified CAR cassette was inserted into the parental vector via *NcoI/XhoI*. The extracellular CAR-binding domain was substituted via *SfiI/NotI*. DARPin-H2C3 (negative control CAR, nc) [12] and CAR containing anti-CD30scFv-binding domain [13] were used as controls. CARs containing CD4-specific scFv-binding domains A8, A12, C1 and G2 were used as benchmark to assess surface expression and specific killing of DARPin_57.2 compared to scFv domains targeting CD4 antigen. CD4-specific scFv antibodies SH1954-A8, -A12, -C1 and -G2 were selected from the human naive antibody gene libraries HAL9/10 (PMID: 25888378) using recombinant CD4. These scFvs were kindly provided by Prof. Dr. Michael Hust, TU Braunschweig. CD4-specific DARPin_57.2 was used with kind permission of Prof. Dr. A. Trkola, ETH Zurich [11].

### Cells and media

HEK293T/17 (CRL-11268, ATCC) cells were maintained in DMEM (Lonza) supplemented with 10% (v/v) heat-inactivated FCS (fetal calf serum, Biochrom) and 2 mM l-glutamine. Raji (CCL-86, ATCC) and J-Lat_8.4 (NIH 9847, obtained through the NIH AIDS Reagent Program, Division of AIDS, NIAID, NIH: J-Lat Full Length Clone 8.4 from Dr. Eric Verdin) cells were cultivated in RPMI1640 medium (Lonza) containing 10% (v/v) FCS and 2 mM l-glutamine. The same medium was applied for human primary T cells, but with additional 200 U/ml penicillin/streptomycin (Carl Roth; T cell medium). RPMI1640 used for cell line HuT78 (TIB-161, ATCC) contained 20% (v/v) FCS and 2 mM l-glutamine. All cell lines were tested and negative for mycoplasma.

### Vector generation and transduction of human T cells

**Fig. 1:**
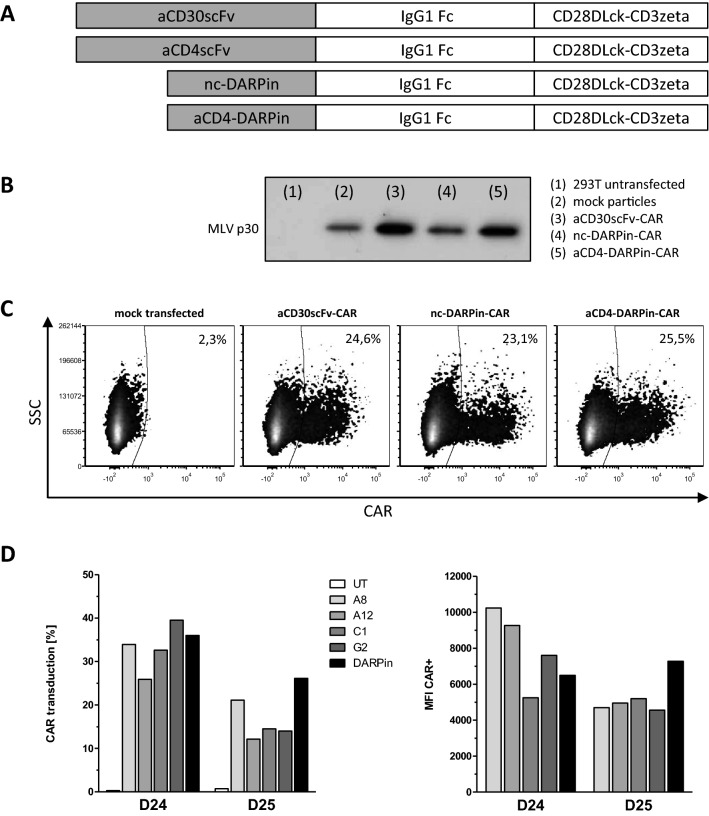
Generation of CAR T cells. **a** Schematic representation of the CAR constructs containing an antigen recognition domain (scFv or DARPin), IgG1 hinge-CH2/CH3 domain, CD28 transmembrane- and intracellular domain, intracellular CD3ζ domain. nc-DARPin is of irrelevant specificity and served as control. **b** Presence of MLV-gag protein p30 as part of γ-retroviral vectors in supernatant of transfected HEK293T/17 cells as detected by Western blot analysis using and MLV p30 specific antibody: mock (lane2) or CAR delivering vector particles (lanes 3–5), supernatant of non-transfected cells served as negative control (lane 1). **c** CAR expression on the surface of CD3^+^ human T cells as recorded by flow cytometry, 3 days post-transduction. **d** Transduction efficiency (left) and Median Fluorescence Intensity (MFI, right) of CD4-specific scFv CARs A8, A12, C1, G2 and DARPin CAR by CD8^+^ T cells of the unrelated donors D24 and D25 as assessed by flow cytometry 12 h post-transduction

**Fig. 2:**
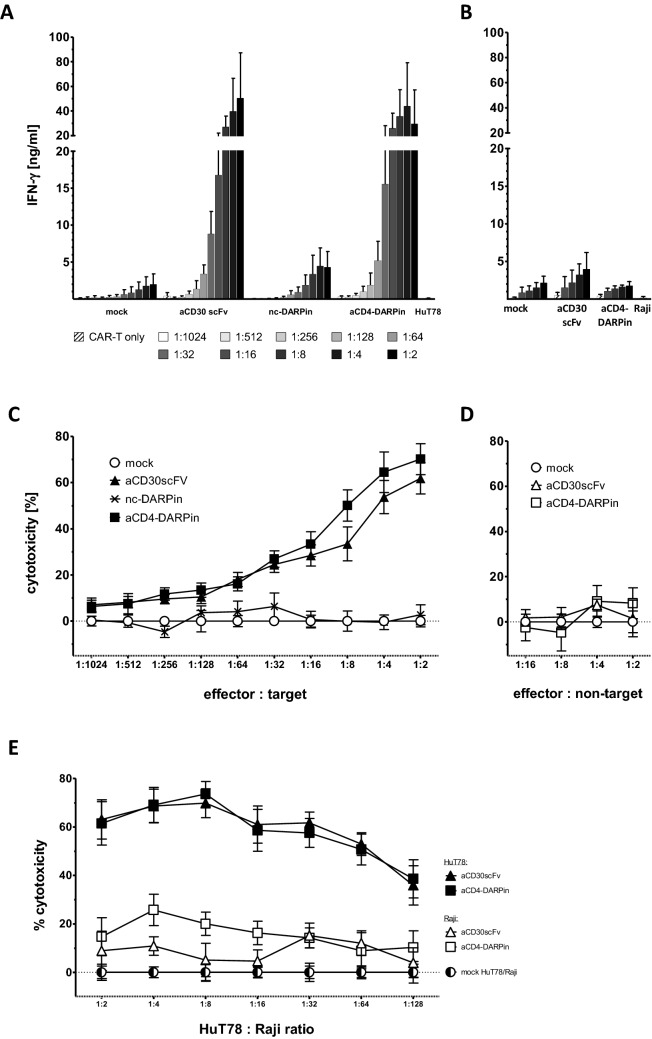
Dose-dependent activation of DARPin CAR T cells and depletion of heterologous target cells after 48 h of co-cultivation. **a**, **b** CAR T cell activation after 48 h co-cultivation with either target (HuT78, A) or non-target (Raji, B) cells was determined by recording secreted IFN-γ in the culture supernatant by ELISA. **c**, **d** The number of remaining HuT78 target and Raji non-target cells was determined by flow cytometry and cytotoxicity [%] calculated in comparison to the mock transduced negative control after the background of antigen-independent killing was subtracted. **e** Specific depletion of CD4^+^ cells after 48 h simultaneous co-cultivation of Hut78, Raji and CAR T cells. CAR T cells were added at a ratio of 1:8 effector to target cells. The percentage of HuT78 cells within the Hut78:Raji mix ranged from 0.78 to 50%. Error bars represent standard error of the mean (SEM, *n* = 6 donors, three independent experiments). In the described experiments, CD4^+^ cells were depleted from the effector CAR T cell population prior to transduction

**Fig. 3:**
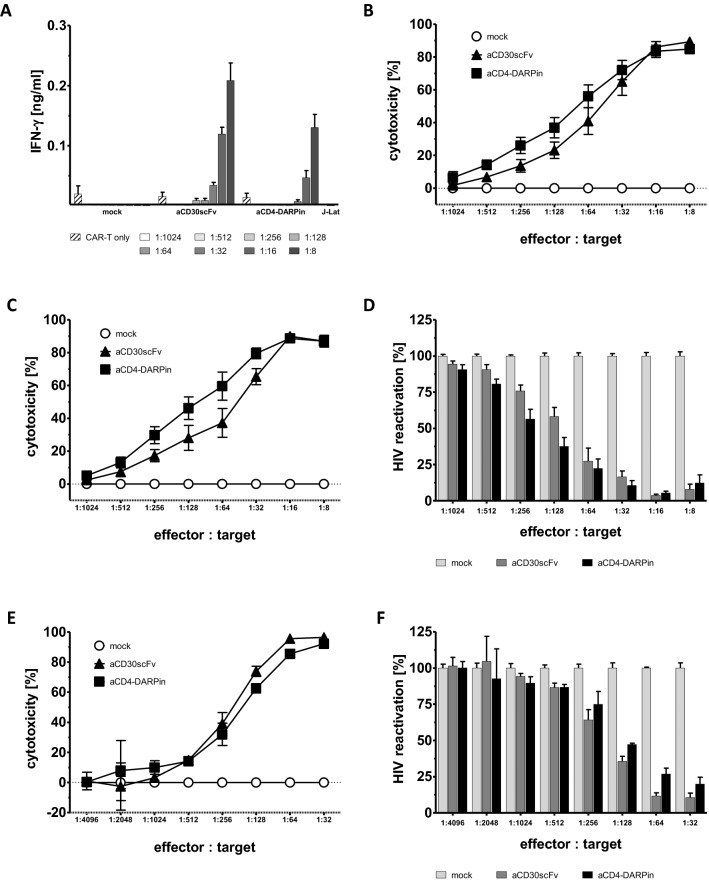
Dose-dependent depletion of HIV^+^ J-Lat cells. **a** CAR T cell activation after 48 h co-incubation with J-Lat cells was determined by recording IFN-γ in the culture supernatant. **b** Number of remaining J-Lat cells after 48 h was determined by flow cytometry. Cytotoxicity [%] was calculated as reduction normalized to mock control. **c**, **d** After 48 h of co-cultivation, GFP expression in J-Lat cells was activated by induction of the HIV promotor with Prostatin and SAHA for 36 h to induce the expression of HIV-eGFP. Total number of J-Lat cells was determined by flow cytometry (**c**), as well as the number of activated HIV-eGFP expressing cells (**d**). **e** Cytotoxicity [%] against J-Lat cells in total, after pre-activation for 36 h followed by 48 h co-culture with CAR T cells. **f** Remaining activated J-Lat cells after 36 h activation prior to 48 h of co-cultivation with CAR T cells. Assays were performed in biological duplicates with cells from *n* = 6 donors in three independent experiments. Error bars represent standard error of the mean (SEM). In the described experiments, CD4^+^ cells were depleted from the effector T cell population prior to transduction

**Fig. 4:**
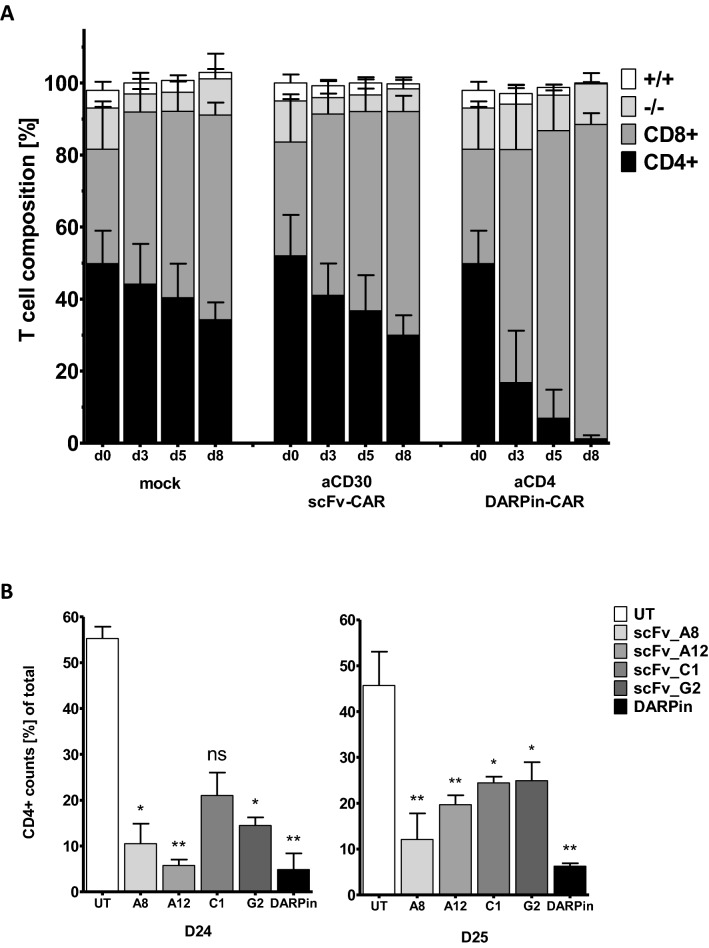
Killing of autologous CD4^+^ T cells. **a** Composition of T cell subsets from peripheral blood after incubation with autologous anti-CD4-DARPin CAR T cells. CD3^+^ T cells were analyzed by flow cytometry for the presence of CD4 (black) and CD8 (grey) on days 0, 3, 5 and 8 post-transduction with MLV-based vectors encoding the anti-CD30scFv CAR, anti-CD4-DARPin CAR or with empty vectors (mock). Data represent the standard deviation of the mean (SD), *n* = 6 donors, three independent experiments. **b** Counts in % of total of autologous CD4^+^ target T cells after 12 h co-culture with CD8^+^ T cells engineered with the CD4-specific scFv or DARPin CAR T effector cells. Data were assessed by flow cytometry showing mean SD of technical triplicates. For normalization, CD8^+^ T cell counts were set to 100% for each sample. Error bars represent mean SD of biological triplicates. Statistical significance compared to untransduced sample (UT) is shown by unpaired student’s *t* test for donors D24 (left; *p ≤ 0.0001, **p ≤ 0.00001) and D25 (right; *p ≤ 0.03, **p ≤ 0.003)

Gammaretroviral transduction of human T cells was performed by spinfection as previously described [14], with minor adjustments. Briefly, retroviral vectors were produced by co-transfection of HEK293T/17 cells with the gammaretroviral vector encoding the CAR or pcDNA3.1 as negative control, helper plasmid pHIT60 encoding for MLV-gag-pol and helper plasmid pcDNA_GaLVwt encoding the gibbon ape leukemia virus (GaLV) envelope protein [15]. Supernatants were harvested 48 h post-transfection, filtered through sterile 0.45 µm PES (polyether sulfone) membrane and stored at − 80 ℃ until further use. For production of scFv-containing CAR T cells, supernatants were harvested 24 h and 48 h post-transfection and used for spinfection of activated T cells. Production of retroviral particles was confirmed by Western blot using goat antiserum against Rauscher Murine Leukemia Virus p30 (ATCC, VR1564ASGt) as detection antibody. For T cell activation, six-well plates (Corning) were coated at 4 ℃ with 1 µg/ml anti-human CD3 (OKT-3, Janssen-Cilag) and 1 µg/ml anti-human CD28 (15E8, Miltenyi) antibody overnight. The next day, peripheral blood mononuclear cells (PBMCs) were isolated from buffy coats using standard Ficoll density gradient centrifugation procedures. Prior to activation, isolated PBMCs were treated as required for the anticipated assay. For heterologous killing of cell lines (Figs. 2 and 3), PBMCs were depleted of CD4^+^ cells using magnetic cell sorting (CD4 MicroBeads, 130–045-101, Miltenyi). For comparison of CAR T cells expressing CD4-specific scFvs or DARPin_57.2, CD4^+^ and CD8^+^ T cells were isolated and activated separately (CD4^+^ T Cell Isolation Kit, 130–096-533 and CD8^+^ T Cell Isolation Kit 130–096-495; Miltenyi) (Fig. 1d, 4b), while for kinetics of autologous T cells over 8 days, PBMCs were activated and transduced, including CD4^+^ and CD8^+^ populations (Fig. 4). PBMCs or T cells were cultivated in the pre-coated plates in T cell medium with additional 500 U/ml ProleukinS (Novartis) for 5 days. For transduction, 5 × 10^5^ cells per well in 24-well plates pre-coated with poly-l-lysine (PLL, Sigma) in 250 µl concentrated medium (55% (v/v) FCS, 40% (v/v) RPMI 1640 medium, 2 mM L-Q, 5% (v/v) penicillin/streptomycin, 2500 U/ml ProleukinS, 24 µg/ml Protamine sulfate (Sigma)), were topped up with 1 ml vector supernatant to a final concentration of 10% (v/v) FCS, 2 mM l-glutamine and 1% penicillin/streptomycin. Plates were spin-occulated at 800 × g, 32 ℃ for 90 min and the transduction procedure was repeated the next day. Three days after the first transduction, CAR surface expression was determined by flow cytometry as described below.

### Killing of heterologous target cells

For heterologous killing assays, isolated PBMCs were depleted of potential CD4^+^ target cells with magnetic beads (Miltenyi) prior to activation and transduction of CD8^+^ T Cells as described above. Subsequently, transduced T cells were washed and cultured for 3 days in T cell medium supplemented with 25 U/ml ProleukinS at 5 × 10^5^ cells per ml. Upon activation, J-Lat_8.4 cells express HIV-R7/E-/GFP (full length HIV-1 minus *env*; GFP (green fluorescent protein) instead of *nef*) [16]. For HIV-1 reactivation, J-Lat_8.4 were cultivated in T cell medium with a final 5 µM Prostatin (Sigma) and 2.5 µM SAHA (suberoylanilide hydroxamic acid, Sigma) for 36 h to induce transcription of GFP under the HIV promotor. CAR T cells were co-cultivated with fluorescently membrane-labeled target (HuT78, J-Lat_8.4) and non-target (Raji) cells in effector to target ratios ranging from 1:8–1:1024 by performing serial dilutions in 96-well round-bottom plates in a total volume of 200 µl per well containing 30,000 target cells. The total number of cells per well was equalized by addition of untransduced autologous T cells. CAR T cells were co-cultivated with target cells for 48 h. Subsequently, the supernatant was removed and stored at − 80 ℃ until subjected to interferon-gamma enzyme-linked immunosorbent assay (IFN-γ ELISA, Mabtech). Heterologous killing was evaluated in flow cytometry by detection of remaining fluorescently labeled target cells (killing [%] = 1 − (average remaining cells pcDNA duplicates/remaining cells sample) × 100).

### Killing of autologous CD4^+^ T cells

Immediately after the second transduction, human CD3^+^ T cells were seeded in 24-well plates at a density of 5 × 10^6^ per well in T cell medium containing 500 U/ml ProleukinS. Duplicate samples from each donor were washed in MACS buffer and fixed in PBS w/o Ca and Mg with 2% (w/v) paraformaldehyde (PFA) on days 0, 3, 5 and 8 post transduction. Subsequently, expression of CARs and cell surface markers CD3, CD4 and CD8 was detected with flow cytometry as described below.

For depletion of autologous T cells by CD4-specific scFv CAR T cells, CD4^+^ and CD8^+^ populations were activated but only the CD8^+^ T cell fraction was transduced as described before. One day post-transduction, cells were rested without IL-2 for 12 h. Subsequently, transduction efficiency of the different CARs was adjusted within the same donor by addition of untransduced T cells and 200,000 (D24) or 50,000 (D25) CD8^+^/CAR^+^ effector cells were co-cultivated with autologous CD4^+^ target cells of the same donor at a 1:1 ratio. Percentage of remaining CD4^+^ T cells was assessed after 12 h by flow cytometry.

### Flow cytometry

Flow cytometric detection of CAR expression on T cells was performed using PE-labeled γ-chain specific F(ab’)2 anti-human IgG antibody (2042–09, Southern Biotech). To distinguish between the different cell types in killing assays with flow cytometry, membranes of target cells were labeled with CellTrace™ Cell Proliferation Kits containing CFSE (HuT78, Raji) or violet (Raji, J-Lat_8.4) dyes, according to the manufacturer’s instructions (Life Technologies) prior to assay setup. Antigen-expression on target cells was detected by staining for anti-human-CD4-PE (SK3, BD Biosciences) and CD30-PE (Ki-2, Miltenyi). Expression on autologous T cells was detected by staining for anti-human-CD4-FITC (RPA-T4-FITC, BD) or -APC (M-T466, Miltenyi), CD3-PE (10D12-PE, Miltenyi), and CD8-APC (IM2469-APC, Beckman Coulter) or CD8-FITC (BW/135/80, Miltenyi) and respective mouse IgG1 isotype controls (BD).

## Results

### Generation of an anti-CD4 CAR containing a DARPin derived binding domain

To engineer DARPin and scFv CARs, we substituted the anti-CD30scFv by other binding domains (Fig. 1a). We inserted DARPins either targeting human CD4 (DARPin_57.2) or a random sequence DARPin not binding to human PBMCs (DARPin_H2C3 (nc-DARPin; supplementary Fig.1) or a CD4-specific scFv. We compared DARPin CAR encoding vector particle production and the efficiency in T cell transduction with CARs based on scFvs [13]. Generation of MLV-based retroviral particles was confirmed by Western blot analyses (Fig. 1b). After transduction, the CARs were expressed on the T cell surface (Fig. 1c, d) indicating that the CD4-DARPin CAR can be likewise expressed by human T cells with similar efficiency as a scFv-based CAR.

### Anti-CD4 DARPin-CAR T cells are specifically activated upon binding of CD4 and deplete target cells in a dose-dependent manner

Cutaneous T lymphoma HuT78 express CD4 and CD30 at high levels; Raji B lymphoma cells are negative for both antigens (Supplementary Fig. 2). To investigate specific activation and cytotoxicity upon antigen binding, we co-cultivated T cells engineered with the CD4-DARPin, CD30scFv, mock (transduced with an empty vector) or nc-CAR, respectively, with HuT78 target or Raji non-target cells. Both, CD4-DARPin and CD30scFv-CAR T cells, were activated upon antigen engagement as, determined by IFN-γ release, dependent on the effector to target cell ratio (Fig. 2a); no activation occurred upon cultivation with Raji cells lacking the cognate antigens (Fig. 2b). Furthermore, mock T cells and control H2C3-DARPin CAR T cells did not secrete IFN-γ above the detection limit (2 pg/ml) of the assay upon co-cultivation with target and non-target cells. Accordingly, T cells with the anti-CD4-DARPin CAR or anti-CD30 scFv CAR-mediated cytotoxicity towards HuT78 cells in a dose-dependent response, whereas control T cells expressing the DARPin-H2C3 CAR or mock T cells did not (Fig. 2c). All CAR T cells and mock T cells had no cytotoxic effect on Raji cells (Fig. 2d). To address the specific elimination of target cells within a mixed cell population, HuT78 and Raji cells were co-cultivated with CAR T cells. Anti-CD4-DARPin CAR T cells specifically depleted the HuT78 target cells from the mixed population, while the number of Raji cells was not altered (Fig. 2e). We concluded that the anti-CD4-DARPin CAR mediates specific T cell activation towards cognate CD4^+^ target cells in vitro.

### DARPin CAR T cells deplete HIV^+^ target cells

J-Lat cells were derived from Jurkat cells by viral infection with a HIV-R7/E-/GFP (full-length HIV 1 minus env, minus nef) retroviral construct, secreting defective viral particles. J-Lat cells latently express GFP, rendering them suitable to study HIV latency and reactivation, and express both CD4 and CD30 that are used as target antigens (Supplementary Fig. 2). J-Lat cells activated CAR T cells with respect to IFN-γ release (Fig. 3a) and were depleted by CAR T cells in a dose-dependent manner, with > 80% cytotoxicity at an effector to target ratio of 1:16 (Fig. 3b, c) or 1:32 (Fig. 3e). Following activation of the HIV promoter to induce GFP expression either before (Fig. 3e, f) or after (Fig. 3c, d) co-cultivation with CAR T cells, the amount of reactivated, GFP-expressing cells was decreased to less than 20% at a ratio of 1:32 effector-to-target cells (Fig. 3d, f, respectively). Data demonstrate a significant reduction of latently HIV-infected cells after 48 h in the presence of DARPin CAR T cells.

### Specific depletion of autologous CD4^+^ cells by DARPin CAR T cells

We investigated whether anti-CD4-DARPin CAR T cells efficiently depleted autologous CD4^+^ cells. For this purpose, we monitored the proportion of CD4^+^ and CD8^+^ cells within the population of human T cells after co-incubation with autologous PBMCs engineered with the CD4-DARPin CAR for 8 days (Fig. 4a; Supplementary Fig. 3). After transduction, the proportion of CD4^+^ cells decreased steadily in the population of cells expressing anti-CD4-DARPin CAR (60% on day 0; 1.3% on day 8). In contrast to that, no significant decrease was observed in the populations containing mock transduced and CD30scFv CAR T cells. Additionally, we compared depletion of primary CD4^+^ T cells by autologous CD8^+^ effector T cells expressing CARs with CD4-specific scFv-binding domains (A8, A12, C1, G2) and DARPin_57.2 (Fig. 4B). For both T cell donors (D24 left, D25 right), anti-CD4-DARPin CAR reduced CD4^+^ target cells significantly when compared to untransduced mock control. Anti-CD4-DARPin CAR T cell killing is at least as efficient as the four anti-CD4scFv-CARs tested in both donors suggesting equally potent activity of the DARPin targeting CAR in comparison to scFv targeting CARs.

## Discussion

One of the major challenges for curing HIV is the elimination of the reservoir of latently infected cells. CAR T cells targeting viral proteins have been investigated for the treatment of HIV infection [17]; however, the latent virus reservoir cannot be completely eliminated by this strategy [18, 19]. Since CD4 is the entry receptor for HIV [20], we sought to eliminate CD4^+^ T cells using a DARPin targeted CAR as a proof-of-concept for complete HIV eradication.

Due to their high stability [21], DARPins appear to be a very promising choice as binding domains for CARs; for example, various lymphocyte receptors have been used for targeting in the context of CARs [22, 23]. We here used an anti-CD4-DARPin [11] for targeting CD4^+^ cells which is well characterized and targets human CD4 [24]. CD4-DARPin CAR T cells were specifically activated upon target recognition and depleted both, heterologous CD4^+^ cells expressing high and low levels of CD4 (HuT78 and J-Lat cells, respectively) and autologous CD4^+^ cells in vitro. The decrease in CD4^+^ cell numbers was not due to a masking-effect of the CD4-binding site by the DARPin-CAR, since the remaining autologous cells were predominantly CD8^+^ T cells.

The anti-CD4-DARPin CAR T cells are aiming at eliminating CD4^+^ cells that are the reservoir of latently HIV-infected cells. The two most pressing questions when targeting and depleting CD4^+^ cells are what side-effects are caused by the depletion of the entire CD4^+^ cell population and if the mature CD4^+^ T cell population can sufficiently recover from naïve stem cells after successful treatment. As an example for recovery after loss of immune cells during CD19 CAR T cell therapy, it was demonstrated that loss of CD19^+^ B cells was well manageable and recovered in most cases [25]. Depletion of the entire CD4^+^ T cell population would leave patients severely immunocompromised; short-term immunodeficiency can be clinically managed as shown, for example, for patients with SCID (severe combined immunodeficiency), a severe dysfunction of B cells, T cells or both [26]. In comparison, with the depletion of the entire CD4^+^ T cell pool, a major part of the immune system, such as innate immunity, B cells, dendritic cells and CD8^+^ effector T cells are expected to remain during treatment; however, without CD4^+^ T cell help. We think that there is a realistic chance that, under appropriate medical care, short-term depletion of CD4^+^ cells will be tolerated. Our assumption is also based on the observation that depletion of CD4^+^ T cells with monoclonal antibodies in non-human primates was safe in previous studies [27], although this did not result in complete eradication of CD4^+^ cell pools. Following complete eradication, CD4^+^ T cells would need to recover from the stem cell pool of treated patients; thus, to terminate therapy, CAR T cells need to be eliminated entirely. Multiple options to eliminate or to switch off CAR T cells are currently under investigation, such as inducible CARs, switch on/off technologies [28–30] or specific depletion using monoclonal antibodies [31]. Upon depletion of CD4^+^ T cells below 5% of total blood cells using monoclonal antibodies in chimpanzees, no serious infections were observed and CD4^+^ T cells recovered within 3 weeks after initial treatment [27, 32]. Subsequent transplantation of hematopoietic stem cells leads to a good prognosis of patient survival [33]. Thus, transplantation of CD34^+^ stem cells is a further treatment option for CAR T cell-treated patients failing to sufficiently recover their CD4^+^ T cell population. Potential immunogenicity is another issue widely discussed in the context of CAR-binding domains [34]. Whether DARPins are less immunogenic than scFvs cannot be answered in general and would have to be assessed for each molecule individually as there are also numerous scFvs of low immunogenicity [35]. There is some evidence that DARPins could be less immunogenic than other binding domains due to their presence in circulating red blood cells, likely leading to immune tolerance [36]. Investigation of DARPins in clinical trials showed no evidence of immunogenicity [37, 38] (clinicaltrials.gov: NCT02194426; NCT02462928; NCT02462486), however, sufficient data are yet to be generated.

The risk of on-target/off-tissue toxicity by anti-CD4 CAR T cells is considered very low since CD4 is a unique target expressed mainly by T cells, monocytes and macrophages. One potential low risk regarding on-target/off-tissue toxicity is expected in neuronal cells of the cerebral cortex where CD4 is expressed, although at lower levels than by hematopoietic cells [39]. However, depleting CD4 T cells by monoclonal antibodies did not cause any damage to neuronal tissues in chimpanzees [27]. One option to minimize the risk could be the use of bi-specific CAR T cells that target CD4 but are disabled by an inhibitory CAR upon binding a marker of the neuronal lineage. Alternatively, binding of the target antigen can be controlled by inducible CAR expression [40], allowing a balance between depletion and subsequent recovery of CD4^+^ cells. This concept in improving selectivity in target cell elimination by CAR T cells is currently under investigation [41]. Another hurdle for therapeutic application of CAR T cells is the potential HIV infection of the CAR T cells themselves. Several approaches have been undertaken to prevent CAR T cells from HIV infection; for example, the inhibition of HIV co-receptor CCR5 in CAR T cells zinc finger nucleases [42]. An ideal target for CAR T cell therapy would be a cellular marker specific for cells harboring integrated HIV DNA. Using various strategies, different teams have reported that surface molecules such as CD32a may be enriched in latently infected cells, however, the impact of those data are controversially discussed (reviewed in [43]). Such markers could represent additional and more specific targets for elimination of HIV-infected cells with CAR T cell therapy. However, this in vitro study clearly demonstrates the proof of concept that CD4-specific DARPin CAR T cells can eliminate the CD4^+^ T cell compartment. The results, therefore, imply that anti-CD4-DARPin CAR T cells might have the potential to substantially reduce the latently HIV-infected T cell reservoir in vivo.

## Electronic supplementary material

Below is the link to the electronic supplementary material.

Supplementary Fig. 1 Lack of specific binding of nc-DARPin to human PBMCs. Soluble DARPins (nc, left and anti-CD4_H2A4, right) were armed N-terminally with a HA-tag (human influenza hemagglutinin-tag) and detected by a PE-labeled anti-HA antibody by flow cytometry after binding to human PBMCs. Fig. 2 Expression of CAR cognate antigens on the surface of HuT78, J-Lat and Raji cells. Cells were stained with anti-CD4 or anti-CD30 antibodies or the respective isotype control. Flow cytometric analysis of fluorescence intensity of CD4 or CD30 on the cell surface is shown. Fig. 3 Composition of T cell subsets during incubation with autologous CAR T cells. CD3+ T cells were analyzed for the expression of CD4 (lower right quadrant) and CD8 (upper left quadrant) on days 0, 3, 5 and 8 post-transduction with vectors encoding the anti-CD30scFv CAR, anti-CD4-DARPin CAR or empty vectors (mock control). One representative donor out of 6, summarized in Fig. 4A is shown. (PPTX 1115 kb)
