# Supplementary material for: Designed Ankyrin Repeat Protein (DARPin) to target chimeric antigen receptor (CAR)-redirected T cells towards CD4+ T cells to reduce the latent HIV+ cell reservoir
Source: Med Microbiol Immunol. 2020 Sep 12;209(6):681–91. doi: 10.1007/s00430-020-00692-0 (PMC7568711; doi:10.1007/s00430-020-00692-0)
Supplement: Supplementary file 1 — Supplementary Fig. 1 Lack of specific binding of nc-DARPin to human PBMCs. Soluble DARPins (nc, left and anti-CD4_H2A4, right) were armed N-terminally with a HA-tag (human influenza hemagglutinin-tag) and detected by a PE-labeled anti-HA antibody by flow cytometry after binding to human PBMCs. Fig. 2 Expression of CAR cognate antigens on the surface of HuT78, J-Lat and Raji cells. Cells were stained with anti-CD4 or anti-CD30 antibodies or the respective isotype control. Flow cytometric analysis of fluorescence intensity of CD4 or CD30 on the cell surface is shown. Fig. 3 Composition of T cell subsets during incubation with autologous CAR T cells. CD3+ T cells were analyzed for the expression of CD4 (lower right quadrant) and CD8 (upper left quadrant) on days 0, 3, 5 and 8 post-transduction with vectors encoding the anti-CD30scFv CAR, anti-CD4-DARPin CAR or empty vectors (mock control). One representative donor out of 6, summarized in Fig. 4A is shown. (PPTX 1115 kb) [file 430_2020_692_MOESM1_ESM.pptx]

## Slide 1
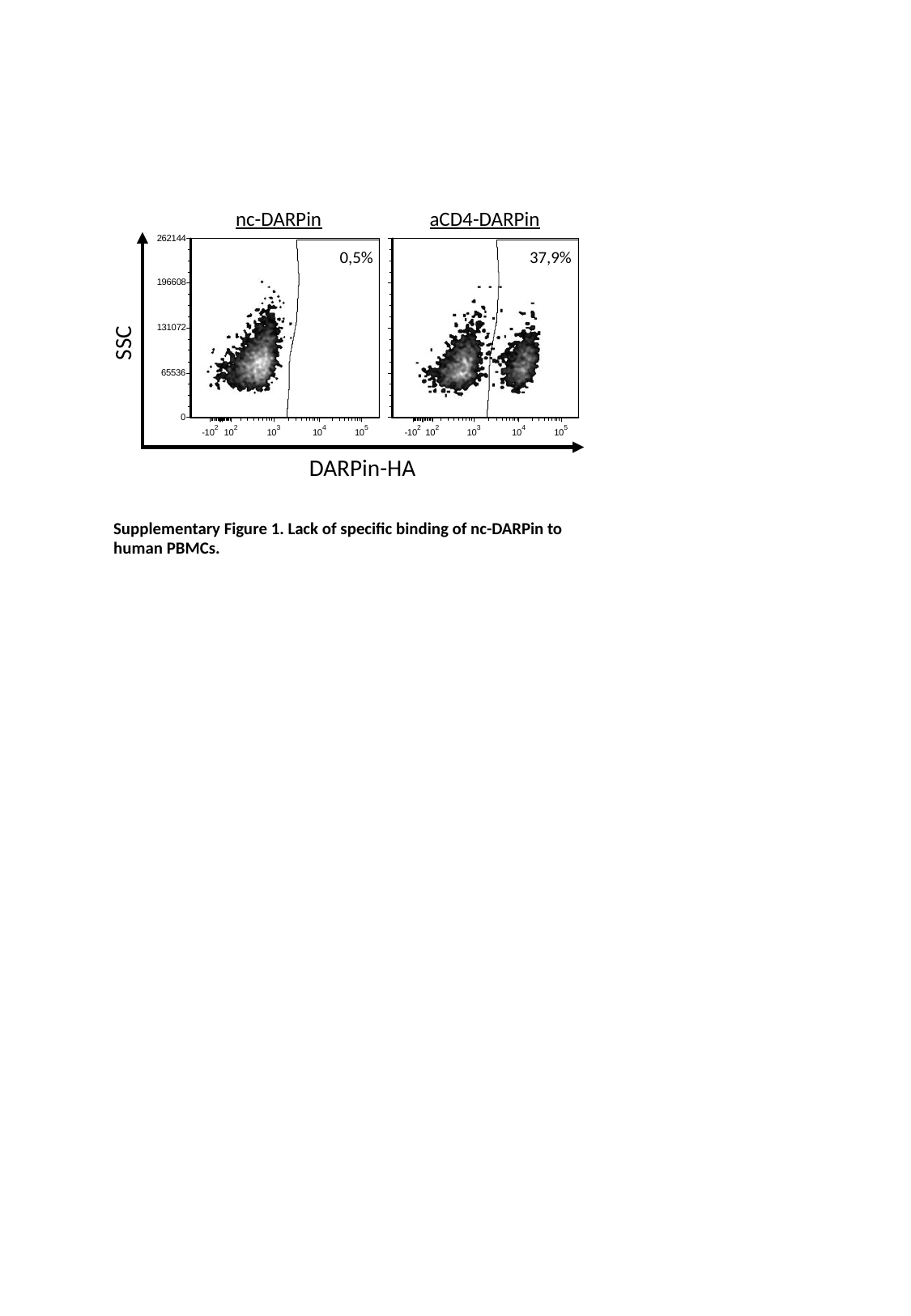

aCD4-DARPin
nc-DARPin
SSC
DARPin-HA
0,5%
37,9%
Supplementary Figure 1. Lack of specific binding of nc-DARPin to human PBMCs.

## Slide 2
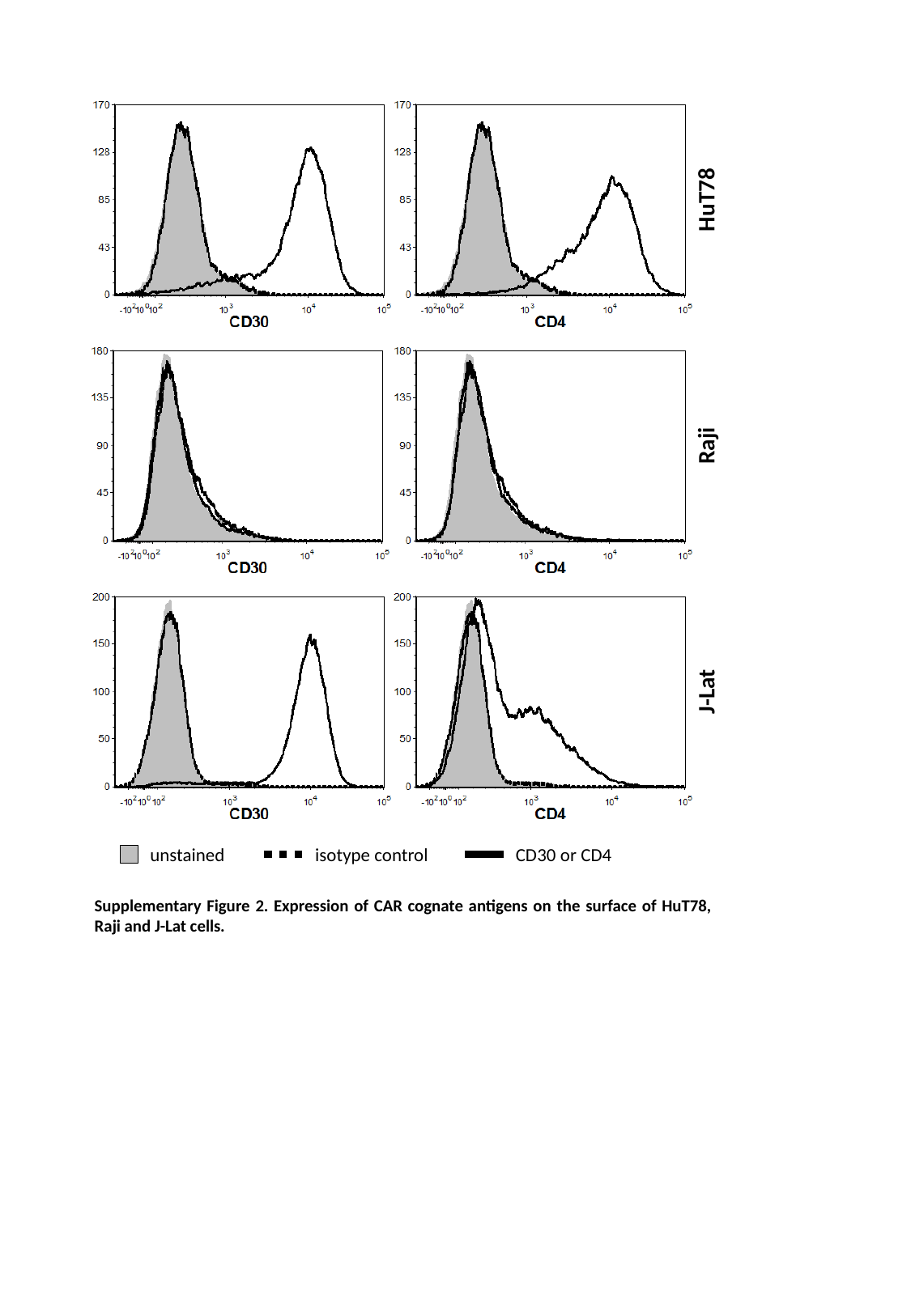

HuT78
Raji
J-Lat
unstained
isotype control
CD30 or CD4
Supplementary Figure 2. Expression of CAR cognate antigens on the surface of HuT78, Raji and J-Lat cells.

## Slide 3
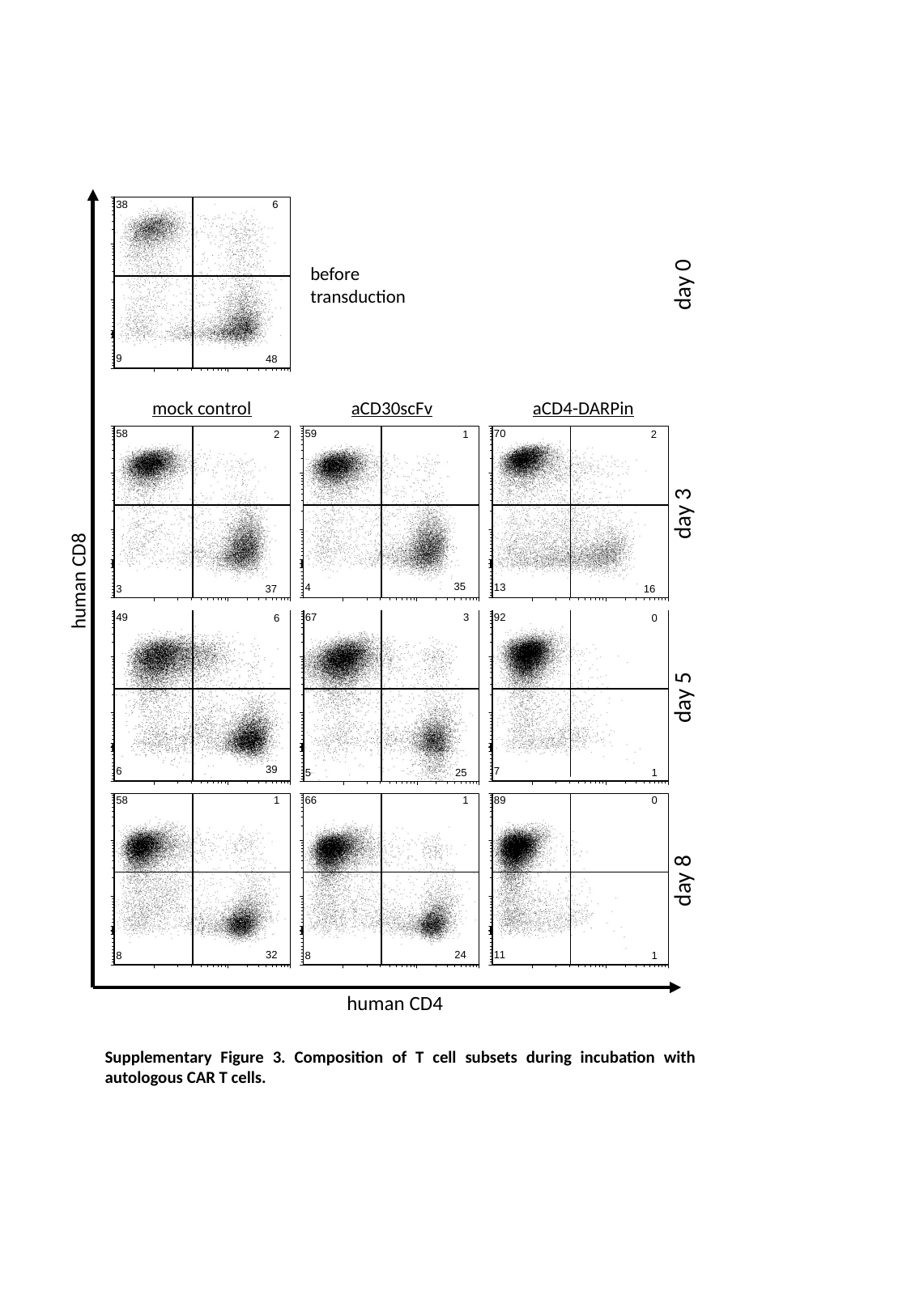

day 0
before
transduction
mock control
aCD30scFv
aCD4-DARPin
day 3
human CD8
day 5
day 8
human CD4
Supplementary Figure 3. Composition of T cell subsets during incubation with autologous CAR T cells.
